# Phomaderide, a unique (6/5/4/5/6) spiro-cyclic dimer from the desert plant endophytic fungus *Phoma betae* A. B. Frank (Didymellaceae)

**DOI:** 10.3389/fchem.2025.1583666

**Published:** 2025-07-07

**Authors:** Hao-Di Sun, Yan-Duo Wang, Hui-Qi Fang, Jian Yang, Yu-Tong Hua, Gang Ding, Lan-Ping Guo

**Affiliations:** ^1^ State Key Laboratory for Quality Ensurance and Sustainable Use of Dao-di Herbs, Institute of Chinese Materia Medica China Academy of Chinese Medical Sciences, China Academy of Chinese Medical Sciences, Beijing, China; ^2^ State Key Laboratory of Bioactive Substance and Function of Natural Medicines, Institute of Medicinal Plant Development, Chinese Academy of Medical Sciences and Peking Union Medical College, Beijing, China; ^3^ State Key Laboratory for Quality Ensurance and Sustainable Use of Dao-di Herbs, National Resource Center for Chinese Materia Medica, China Academy of Chinese Medical Sciences, Beijing, China

**Keywords:** phomaderide, desert plant endophytic fungus, building block-based molecular network, cytotoxicity, structural elucidation, natural products

## Abstract

**Introduction:**

Endophytic fungi from desert plants are prolific producers of structurally unique stress-responsive metabolites. This study investigates the secondary metabolites of Phoma betae A. B. Frank (Didymellaceae), a desert plant endophytic fungus, aiming to discover novel bioactive compounds through advanced molecular networking strategies.

**Methods:**

A building blocks-based molecular network (BBMN) strategy was employed to screen the fungal extract. Target compounds were isolated using silica gel and ODS column chromatography, followed by semi-preparative HPLC purification. Structural elucidation was achieved through comprehensive NMR spectroscopy, mass fragmentation pathway analysis, and electronic circular dichroism (ECD) calculations. Cytotoxicity was evaluated against HeLa and A549 cancer cell lines using CCK-8 assays.

**Results:**

Three compounds were characterized:Phomaderide (3), a unique (6/5/4/5/6) spiro-cyclic dimer formed via stereoselective [2+2] photocycloaddition of two phaeosphaeride A (1) monomers. Its biosynthetic precursor phaeosphaeride A (1). A new hydroxylated analog, phaeosphaeride C (2). Compounds 2 and 3 exhibited moderate cytotoxicity against HeLa (IC_50_ 29.97–39.15 μM) and A549 cells (IC_50_ 30.47–58.33 μM).

**Discussion:**

This work highlights the metabolic versatility of extremophilic fungi, demonstrating Phoma betae's capacity to generate architecturally complex molecules. Phomaderide's unprecedented spiro-cyclic dimer scaffold positions it as a promising lead for anticancer drug discovery, with structural modifications (hydroxylation and dimerization) significantly influencing bioactivity. The BBMN strategy proved effective for targeted isolation of structurally related analogs from complex extracts.

## 1 Introduction

Endophytic fungi of desert plants, under extreme environmental stress, frequently biosynthesize structurally unique stress-responsive metabolites. During the long-term evolutionary adaptation to these environments, these fungi have produced a variety of secondary metabolites that are a promising resource of biologically active natural products (Li et al., 2020; Xu et al., 2022; Tan Y. et al., 2022; Zhang et al., 2021; Tan et al., 2019; Tan X. M. et al., 2022). In our prior research, a series of undescribed diphenyl ethers were isolated from the endophytic fungus *Phoma betae* A. B. Frank (Didymellaceae) inhabiting a desert plant, and an empirical rule about the shielding effects induced by an aromatic ring in diphenyl ethers was first observed and analyzed (Tan Y. et al., 2022; Liu et al., 2023).

In a previous study, ten diphenyl ethers (DPEs) were isolated from the desert plant endophytic fungus *P. betae* A. B. Frank (Didymellaceae) (Tan Y. et al., 2022). Bioactivity evaluation revealed moderate cytotoxicity and potent antioxidant activities, highlighting their potential as antioxidant agents.

In the continued exploration of secondary metabolites with novel structures from this fungus, phaeosphaeride A (**1**) was isolated from the endophytic fungus *Phaeosphaeria avenaria*. This metabolite, with a unique ring system, exhibited strong inhibitory activity of signal transducer and activator of transcription **3** (STAT3) (Maloney et al., 2006). Its unique ring system and excellent bioactivities attracted different groups for total synthesis, which established its correct relative and absolute configurations (Chatzimpaloglou et al., 2012; Chatzimpalogou et al., 2014; Kobayashi et al., 2015). The building block-based molecular network (BBMN) method integrates molecular networking with the concept of building blocks (He et al., 2021; Zhu et al., 2023). By extracting fragments containing characteristic fragment ions or neutral losses from MS^2^ spectra, this approach simplifies large amounts of raw data and generates more targeted molecular network results.

**FIGURE 1:**
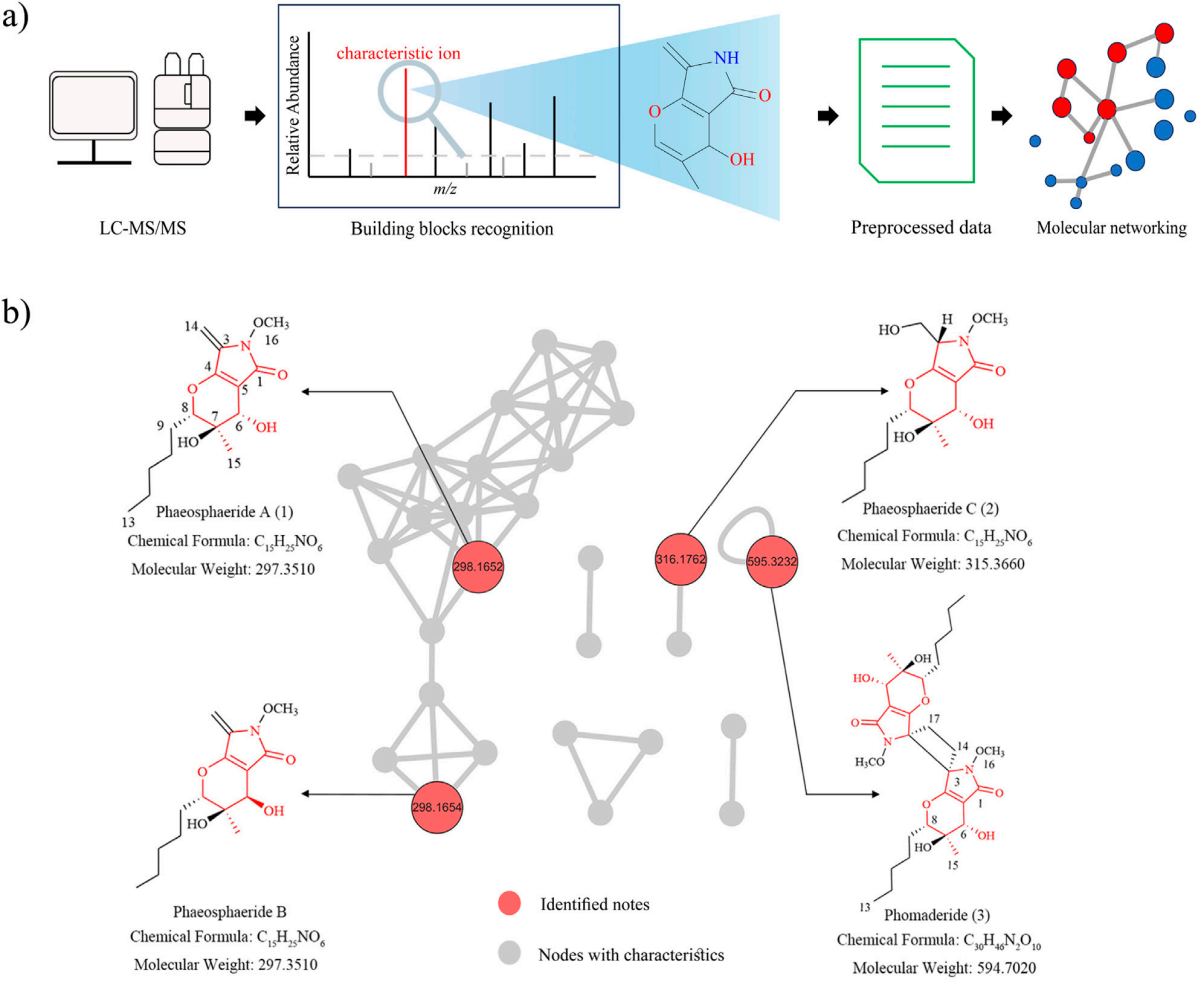
**(A)** Schematic workflow of the application of the building block-based molecular network (BBMN). **(B)** BBMN of the spectra with the refined features.

In this study, a BBMN strategy was employed to rapidly screen analogs phaeosphaeride C (**2**) and phomaderide (**3**) related to phaeosphaeride A (**1**) from the extract of *P. betae* A. B. Frank (Didymellaceae) of these compounds (Figure 1). Phomaderide (**3**) is a structurally unprecedented (6/5/4/5/6) spiro-cyclic dimer formed via a [2 + 2] photocycloaddition of two phaeosphaeride A monomers. Its complex architecture includes a cyclobutane core, multiple stereocenters, and fused heterocyclic systems, reflecting significant structural divergence. While DPEs primarily exhibited antioxidant properties, phomaderide and its precursor analog phaeosphaeride C (**2**) demonstrated moderate cytotoxicity against HeLa and A549 cells, suggesting distinct bioactive mechanisms. This comparison underscores the metabolic versatility of *P. betae* A. B. Frank. The isolation, structural elucidation, possible biogenetic pathways, and biological activities of compounds (**1**–**3**) are presented in this study.

## 2 Materials and methods

### 2.1 General experimental procedures

1D and 2D NMR data were recorded on a Bruker AVANCE III 500 MHz (Bruker, Massachusetts, America) or a Varian Inova 600 MHz spectrometer (Varian, California, America), using CDCl_3_ or DMSO-*d*_6_ signals as references (*δ*_H_/*δ*_C_ 7.26/77.2, 2.50/39.5). HR-ESI-MS spectra were obtained on a UPLC-Q-TOF-MS/MS (Waters Xevo G2-s QTOF, United States). In addition, optical rotations were obtained on a 241 polarimeter (PerkinElmer, Waltham, America), and UV spectra were measured on a UV-2102 (Unico, Shanghai, China) instrument. IR spectra were recorded on an FTIR-8400S spectrophotometer (Shimadzu, Kyoto, Japan), and the J-815 spectropolarimeter (JASCO, Tokyo, Japan) was used for collecting CD spectra. For purification, semi-preparative high-performance liquid chromatography (HPLC) was performed on a SEP LC-52 instrument with an MWD UV detector (Separation (Beijing) Technology Co Ltd., Beijing, China) which was packed with an Octadecylsilyl (ODS) column (YMC-pack ODS-A, 5 μm, 250 × 10 mm, Kyoto, Japan).

### 2.2 Fungal material fermentation and extraction

The strain of *P. betae* A. B. Frank (Didymellaceae) was isolated from the desert plant and provided by the Chinese Academy of Agricultural Sciences.

*Phoma betae* A. B. Frank was cultured on PDA plates at 25°C for 7 days. Then, the cells were transformed into a sterilized solid medium containing rice (60.0 g) and distilled water (80 mL) in Fernbach flasks (500 mL) for further fermentation at 25°C for 30 days. Finally, the whole fermented material was extracted with ethyl acetate (EtOAc) three times, and the extract was concentrated under reduced pressure to yield 150 g of the crude extract.

### 2.3 Purification of structures

The original extract was fractionated on a silica gel column chromatograph using dichloromethane (CH_2_Cl_2_) ether and methanol (MeOH) (1:0–0:1, each 5.0 L) to obtain nine fractions (Fr.C1–Fr.C9). Fr.C2 (0.56 g) was separated on an ODS column chromatography with MeOH in H_2_O (20:80–80:20, each 260 mL) to get seven subfractions. Fr.C2.3 (39.3 mg) was further purified by semi-preparative HPLC (58% MeOH in H_2_O, v/v, 2 mL/min) to yield **1** (19.9 mg, *t*_R_ 26.1 min). Fr.C3 (2.29 g) was fractionated on an ODS column chromatograph using MeOH in H_2_O elution (30:70–90:10, each 500 mL) to get eight subfractions. Fr.C3.3 (14.4 mg) was further purified by semi-preparative HPLC (60% MeOH in H_2_O, v/v, 2 mL/min) to obtain **2** (2.8 mg, *t*_R_ 12.4 min). Fr.C3.6 (111.4 mg) was further separated by semi-preparative HPLC (78% MeOH in H_2_O, 2 mL/min) to yield **3** (11.5 mg, *t*_R_ 28.7 min).

### 2.4 Preparation of (*R*)- and(*S*)-MTPA esters of compound 2

(*R*)-MTPACl (8 μL) was added to a solution of compound **2** (0.8 mg) in pyridin-*d*_5_ (200 μL) and placed at room temperature for 24 h. The crude product was purified by HPLC (60% acetonitrile in H_2_O, 2 mL/min) to afford (*S*)-MTPA ester (≈0.5 mg, *t*_R_ = 15.4 min). (*S*)-MTPACl (8 μL) was added to a solution of compound **1** (0.5 mg) in pyridin-*d*_5_ (200 μL) and placed at room temperature for 24 h. The crude product was purified by HPLC (60% acetonitrile in H_2_O, 2 mL/min) to afford (*R*)-MTPA ester (≈0.4 mg, *t*_R_ = 18.51 min). ^1^H NMR data from the (*S*)- and (*R*)-MTPA ester derivatives of phaeosphaeride C (**2**) are shown in Supplementary Figure S12.

### 2.5 Identification of building blocks by characteristic fragmentations filtration

The building blocks were identified by comparing the MS data of compound **1** reported in previous studies. A series of fragments with relatively high abundance and stability were observed in the mass spectrum of **1**. After deducing the fragmentation pathway of **1**, the product ions at (m/z) 180.0671 were selected to recognize the building blocks of phaeosphaeride A (**1**) from the LC-MS dataset. The script for product ion and ion intensity filtering was written in Python (version 3.10.2) and ran on Cursor. Considering the quantity limitation of subsequent isolation and structural elucidation procedures, an additional filter was used with the following settings: m/z tolerance = 0.02, minimum peak area = 1.0E4. Additionally, we designed two versions of the extraction tool, allowing users to select the appropriate tool based on the file format of the raw data (https://github.com/mujinyu233/BBMN-extract).

### 2.6 Biological activities of 2 and 3

A549 and HeLa cells were seeded in 96-well plates at a density of 8,000 cells per well and incubated in Dulbecco’s modified Eagle medium (DMEM) for 24 h. After incubation, the cells were treated with compounds **2** and **3** for 48 h. Subsequently, the culture medium was removed, and 100 μL of CCK-8 reagent (diluted 10-fold in DMEM) was added to each well. The plates were further incubated at 37°C for 1 h. Finally, the optical density was measured at 450 nm using a microplate reader. The **2** and **3** stock solutions (100 mM) were prepared with dimethyl sulfoxide (DMSO). The indicated concentrations were prepared immediately before use.

### 2.7 Photo-induced synthetic reactions

A solution of phaeosphaeride A (**1**) (20 mg, 0.17 mmol) in 1,4-dioxane (1.0 mL) was irradiated with a 500-W high-pressure mercury lamp for 6 h. The solvent was evaporated under vacuum, and the residue was purified by semi-preparative HPLC (54% acetonitrile in H_2_O, 2 mL/min) to yield **3** (0.3 mg, tR 26 min).

## 3 Results and discussion

### 3.1 Construction of building block-based molecular networking

UPLC-Q-TOF-MS/MS analysis was first employed to study the secondary metabolites of *P. betae* A. B. Frank (Didymellaceae). Initially, known compounds were annotated based on featured-based molecular networking, leading to the identification of phaeosphaeride A (**1**) from the fungal extract. In order to determine the characteristic fragment ions of BBMN, the protonated parent ion (*m/z* 298) of compound **1** was observed in the UPLC-Q-TOF-MS/MS spectra with a relatively low abundance. The fragmentation routes showed that the fragment ion (*m/z* 280) was produced from the protonated parent ion (*m/z* 298) through the loss of one molecule of H_2_O (−18). Then, the fragment ion (*m/z* 280) undergoes successive neutral losses of a CH_2_O (−30) molecule and a C_5_H_10_ (−70) molecule, resulting in the formation of characteristic fragment ions (*m/z* 180) (Supplementary Figure S1) (corresponding to the pyranopyrrole unit). Hence, the diagnostic ion at (*m/z)* 180 was used to filter the MS^2^ data to construct the BBMN network (Figure 1), and several nodes were selected with significant features (red nodes) for further investigation. Under the guidance of the BBMN strategy, compounds **2** and **3** were subsequently isolated following quick pinpointing. In this study, a Python-coded building blocks extraction tool was developed to filter and identify MS^2^ data containing target ions and output results based on ion intensity.

### 3.2 Structural elucidation of the new compounds

The molecular formula of compound **2** was determined to be C_15_H_25_NO_6_, indicating that there was one more hydroxyl in **2** than in compound **1**. In addition, the molecular formula of compound **3** was determined to be C_30_H_46_N_2_O_10_, which might be a dimeric phaeosphaeride. Phaeosphaeride C (**2**) and phomaderide (**3**) were then isolated from the extract.

Compound **2** was isolated as a yellow oil. Comparison of the NMR data with those of pheosphaeride A (**1**) indicated structural similarities, with key differences that included the disappearance of the exocyclic double bond signal at C-3 and the emergence of a multiplet integrating for one proton in the mid-field region (*δ*_H_ = 4.12). The ^13^C-NMR spectrum displayed a CH signal at *δ*_C_ = 62.6, suggesting structural modification at C-3. Further analysis of heteronuclear multiple bond coherence (HMBC) correlations between H-14 (*δ*_H_ 4.04, 3.90) and C-3 (*δ*_C_ 62.6)/C-14 (*δ*_C_ 58.3), combined with heteronuclear single quantum coherence (HSQC) cross peaks (*δ*_H-14a,b_ = 4.04, 3.90; *δ*_C-14_ = 58.3), confirmed the attachment of a -CH_2_-OH moiety at C-3 (Supplementary Figures S2, S3), which was supported by 2D-NMR spectral data (Supplementary Figures S5, S6). The relative configuration of **2** was the same as that of **1** based on rotating-frame nuclear Overhauser enhancement spectroscopy (ROESY) correlations (Supplementary Figure S7). Weak spatial interactions between 15-Me and 6-OH, as well as between H-8 and H-6, confirmed that **2** shares the same relative configuration as **1**. The stereochemistry of **2** was determined to be 3*R*, 6*S*, 7*R*, and 8*S* according to modified Mosher’s reactions (Supplementary Figures S12, S13).

Phomaderide (**3**) was isolated as a colorless oil, and its molecular formula was assigned as C_30_H_46_N_2_O_10_ through the HR-ESI-MS [M + H]^+^ (*m/z* 595.3267, calculated 595.3231) with nine degrees of unsaturation. The ^1^H and ^13^C spectra of **3** (Table 1) gave only 23 protons and 15 carbons, suggesting that compound **3** might be a symmetrical dimer. The HSQC spectrum of **3** revealed the presence of a carbonyl carbon, two olefinic carbons, two quaternary carbons, two oxygenated methines, five methylenes, two methyl groups, a methoxy group, and two exchangeable protons.

**TABLE 1:** ^1^H and^13^C NMR data (DMSO-*d*_6_) of 3.

No.	*δ*_H_, (*J* in Hz)	*δ*_C_
1		170.3, C
3		70.7, C
4		167.7, C
5		103.7, C
6	3.96, d (6.0)	65.9, CH
7		70.4, C
8	4.05, m	86.0, CH
9	1.71, m	27.8, CH_2_
10	1.55, m 1.39, m	26.2, CH_2_
11	1.30, m	31.2, CH_2_
12	1.30, m	22.0, CH_2_
13	0.87, t (6.5)	13.9, CH_3_
14	2.64, m 1.99, m	24.9, CH_2_
15	1.08, s	18.2, CH_3_
16	3.71, s	65.2, CH_3_
6-OH	5.27, d (6.0)	
7-OH	4.76, d (3.5)	

^a^ Recorded at 500 MHz.

^b^ Recorded at 125 MHz.

**FIGURE 2:**
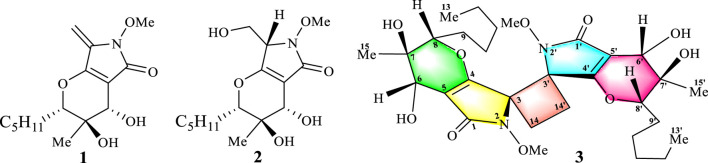
Structures of phaeosphaeride A **(1)**, phaeosphaeride C **(2)**, and phomaderide **(3)**.

**FIGURE 3:**
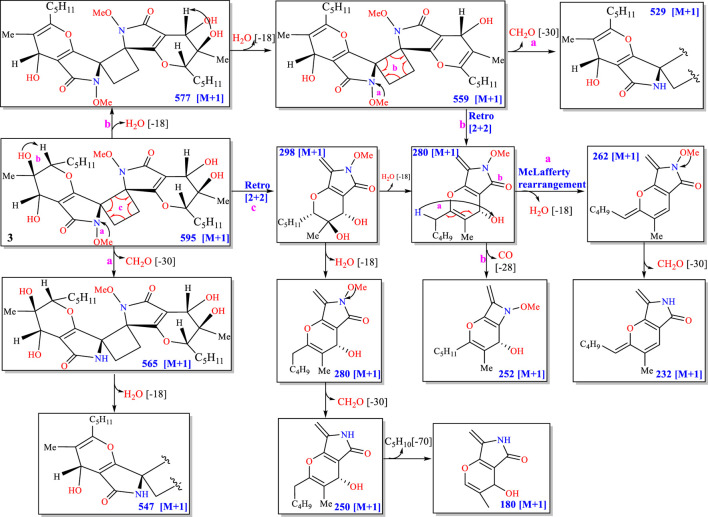
Mass fragmentation pathways of **3**.

The ^1^H–^1^H COSY correlations of **3** gave two isolated fragments corresponding to OH-CH-6 and -CH-8−CH_2_-9−CH_2_-10−CH_2_-11−CH_2_-12−CH_3_-13 (Figure 2), and the remaining connectivity was established by HMBC correlations. The correlations from 15-Me and 7-OH to C-6, C-7, and C-8 supported that C-7 was an oxygenated quaternary carbon connected with C-6, C-8, C-15, and 7-OH. The HMBC correlations of 6-OH with C-5, C-6, and C-7 confirmed the connectivity of C-6 with C-5 and C-7. The cross peaks in the HMBC spectrum from H-6 to C-1, C-4, and C-5 suggested the connection of C-5 with C-1, C-4, and C-6. The correlation from H-8 to C-4 led to an ether linkage between C-4 and C-8 that constructed a 3,4-dihydro-2*H*-pyran ring. The correlations from -CH_2_-14 to C-3 and C-4 determined the connectivity of C-3 with C-4 and C-14. Considering the chemical shift values of the -OMe and C-3 (*δ*_H/C_ = 3.71/65.2; *δ*_C-3_ = 70.7) and similar NMR spectra between **3** and **1**/**2**, the *N*-methoxypyrrolidin-2-one was suggested to exist in **3** the same way it did in **1** and **2**. By accounting for the multiplets (three doublets overlapped in the ^1^H-NMR spectrum) of H-14a and H-14b, a head-to-head and tail-to-tail [2 + 2]-cycloaddition adduct of two identical monomers (**1**) was suggested to be the structure of **3** (Zhang et al., 2016; Shen et al., 1994; Yang et al., 2023). This conclusion was also supported by MS/MS experiments. The mass fragmentation pathways of **3** revealed that a Retro [2 + 2] reaction, a neutral loss (H_2_O, CO, and HCOH), and a McLafferty rearrangement (-H_2_O) were the main cleavage patterns (Figure 3).

**FIGURE 4:**
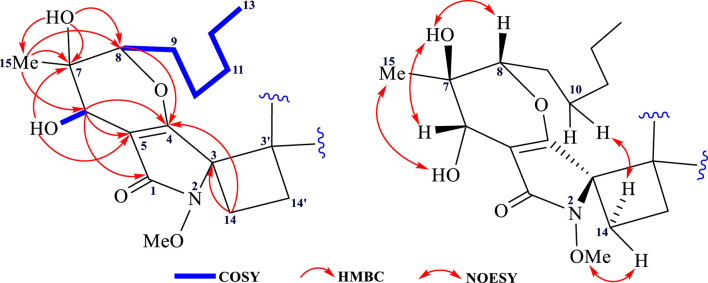
Key 2D-NMR correlations of **3**.

**SCHEME 1:**
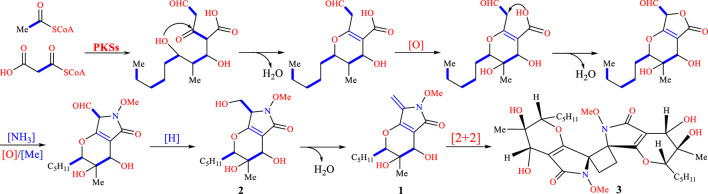
Plausible biosynthetic pathways of **1**–**3**.

The relative configuration of phomaderide (**3**) was determined by the NOESY correlation (Figure 4). The correlations from 7-OH to H-6 and H-8 revealed that these groups were on the same face of the 3,4-dihydro-2*H*-pyran ring, whereas the correlations from 15-Me to 6-OH suggested that the two groups were on the other side of the pyran ring. The weak NOESY correlations from H-14a to -OMe and from H-14b to CH_2_-10- indicated that these protons were close in space. Theoretically, the stereochemistry of **3** was similar to that of **1** and **2** based on their structural features and possible biosynthetic pathways. The proposed biosynthetic pathway for compounds **1**, **2,** and **3** (Scheme 1) aligns with fungal secondary metabolism logic. The N-methoxypyrrolidin-2-one core in **1** likely originates from a hybrid polyketide-amino acid pathway (Miyanaga et al., 2018), with O-methylation at C-3 mediated by enzymes (You et al., 2024). The stereoselective [2 + 2] photocycloaddition forming **3** represents a rare but mechanistically coherent step. While abiotic photodimerization is plausible (supported by photocatalytic experiments), enzymatic mediation via ROS-generating oxidases could enforce stereocontrol, as evidenced by the exclusive isolation of a single stereoisomer. The head-to-head/tail-to-tail dimerization minimizes steric clashes, preserving stereochemistry at C-6/C-8.

**FIGURE 5:**
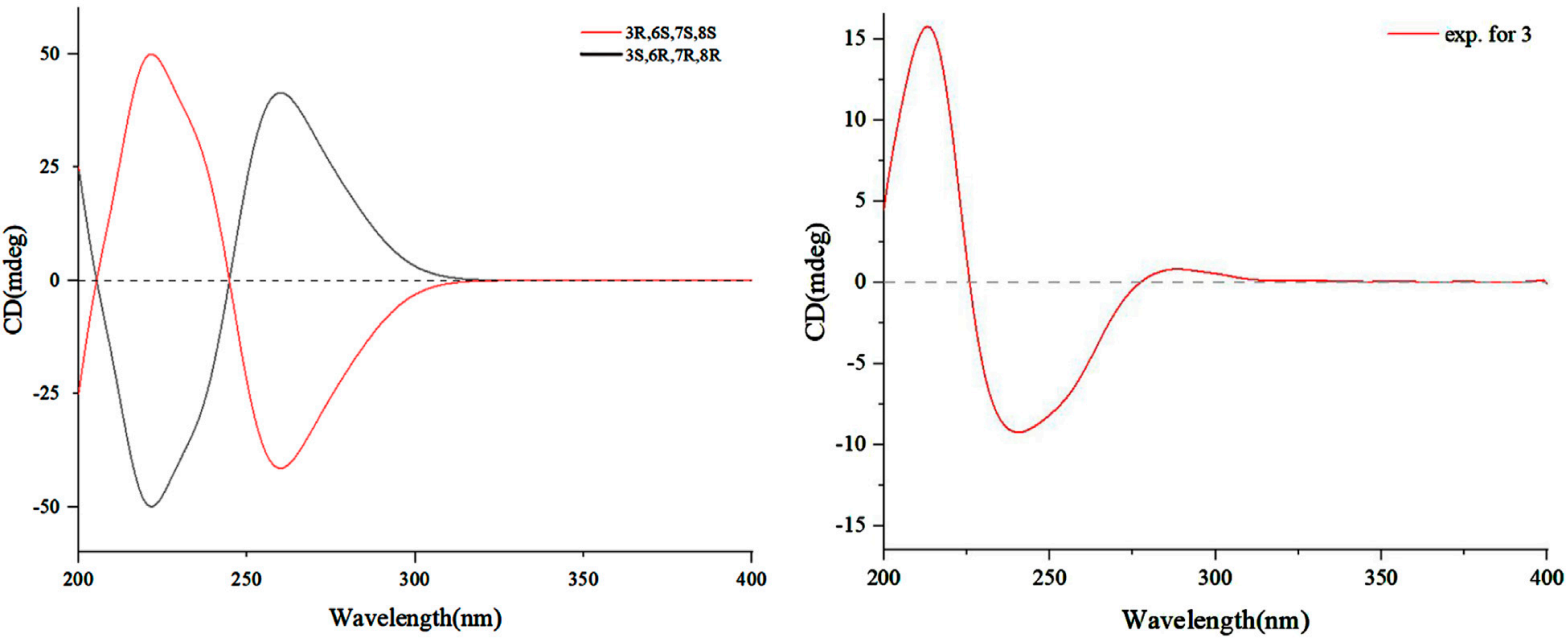
Calculated and experimental ECD spectra of **3**.

Despite coherence, key questions remain: the timing of O-methylation, enzymatic *versus* abiotic dimerization triggers and ecological drivers of dimer selectivity. Resolving these requires gene cluster analysis and isotopic labeling studies. Overall, the pathway exemplifies fungal innovation in merging polyketide logic with radical-mediated cycloadditions, underscoring desert endophytes as reservoirs of structurally unique metabolites shaped by environmental pressures. To further support this hypothesis, modified Mosher’s reactions were tried but were not successful. Thus, the absolute configuration of **3** was determined by the comparison of the ECD spectrum recorded in MeOH and the DT-DFT-calculated spectrum of **3** at the B3LYP16-311+G (d, p) level. The calculated ECD spectrum of **3** matched with the experimental ECD spectrum (Figure 5), which suggested its stereochemistry to be 3*R*, 6*S*, 7*R*, and 8*S*.

To verify whether **3** is an artificial product, a photocatalytic approach was employed to induce the [2 + 2] cycloaddition reaction of **1**. Theoretically, four stereoisomers could be formed considering two chiral carbons at C-3/C-3′. However, natural products possessing cyclobutane skeletons typically exist in only a few predominant stereoisomeric forms, which might be due to stereoselectivity influenced by steric hindrance, electronic effects, *E*/*Z* tautomerization, and ring strain of olefinic bonds (Yang et al., 2023; Sun et al., 2024; Liu and Hu, 2024). Due to the low yield of the products, only the MS analysis was investigated (Supplementary Figure S23), which suggested that biomimetic synthesis yielded a single stereoisomer through light-induced [2 + 2] cycloaddition. Furthermore, phaeosphaeride A (**1**) without dimeric structures was co-isolated in previous reports (Maloney et al., 2006), indicating that **1** could not undergo spontaneous [2 + 2] cycloaddition to shape the dimer phomaderide (**3**). More importantly, the low yield of phomaderide (**3**) in the photocatalytic synthesis implied that this dimer with a unique ring system was not an artifact.

### 3.3 Bioactivity assay

Compound **1** displayed strong inhibitory activity against STAT3 related to oncogeneis and also inhibited the myeloma cell growth, whereas its diastereomer phaeosphaeride B did not show activity against STAT3 (Maloney et al., 2006). Considering the structural similarity, the cytotoxic activities of compounds **2** and **3** were evaluated against several cancer cell lines. Compounds **2** and **3** exhibit moderate inhibitory activity against HeLa cells with half-maximal inhibitory concentration (IC_50_) values ranging from 29.97 μmol/L to 39.15 μmol/L. In addition, compounds **2** and **3** exhibited weak inhibitory activity against non-small cell lung cancer cells A549, with IC_50_ values ranging from 30.47 μ mol/L to 58.33 μ mol/L (Supplementary Tables S2, S3).

## 4 Conclusion

In summary, guided by the BBMN strategy, a chemical investigation was performed on the plant endophytic fungus *P. betae* A. B. Frank (Didymellaceae), which led to the discovery of phomaderide (**3**) with a unique 6/5/4/5/6 pentacyclic system, featuring two spiro rings and multiple heteroatoms. Phomaderide (**3**) is formed by the linkage of two identical precursors through a double bond [2 + 2] cycloaddition, resulting in a cyclobutane structure with a symmetric structure and eight stereocenters. This complex carbon skeleton possesses ten oxygen atoms forming different functional groups, including amide, ether bonds, -OH, and -NOMe, which implies it as a potential star molecule for organic synthesis. The structural modifications of hydroxylation and dimerization exert significant impacts on biological activity. Consistent with prior analogous studies, the hydroxylation at C-14 in compound **2** may enhance hydrogen bonding capacity and lipophilicity (Liu et al., 2025; Kim et al., 2025), providing a theoretical rationale for its moderate cytotoxicity against HeLa cells (IC50 29.97 μM). Conversely, the [2 + 2] dimerization in **3** introduces a strained cyclobutane core and bis-spirocyclic fused N-methoxypyrrolidone systems, resulting in heightened molecular rigidity and polarity. These structural alterations likely compromise membrane permeability (Haruna et al., 2021), thereby accounting for the attenuated cytotoxicity (IC50 39.15 μM). These findings underscore the critical balance between hydrophilicity, molecular topology, and bioactivity in natural products. Our result further implied that fungi from unique bio-environmental fields, such as under-investigated endophytic fungi from desert plants, will be a potent new resource for novel and bioactive secondary metabolites. In future studies, establishing a framework for rational analog design with optimized pharmacokinetic profiles and fully elucidating the mechanistic implications of these structural features will be essential.

## Data Availability

The original contributions presented in the study are included in the article/Supplementary Material; further inquiries can be directed to the corresponding authors.
